# Nitrogen-Doped Diamond Film for Optical Investigation of Hemoglobin Concentration

**DOI:** 10.3390/ma11010109

**Published:** 2018-01-11

**Authors:** Daria Majchrowicz, Monika Kosowska, Kamatchi J. Sankaran, Przemysław Struk, Michał Wąsowicz, Michał Sobaszek, Ken Haenen, Małgorzata Jędrzejewska-Szczerska

**Affiliations:** 1 Department of Metrology and Optoelectronics, Faculty of Electronics, Telecommunications and Informatics, Gdansk University of Technology, 80-233 Gdansk, Poland; nika.kosowska@gmail.com (M.K.); micsobas@pg.gda.pl (M.S.); 2 Institute for Materials Research (IMO), Hasselt University, B-3590 Diepenbeek, Belgium; sankaran.kamatchi@uhasselt.be (K.J.S.); ken.haenen@uhasselt.be (K.H.); 3 IMOMEC, IMEC vzw, B-3590 Diepenbeek, Belgium; 4 Department of Optoelectronics, Faculty of Electrical Engineering, Silesian University of Technology, 44-100 Gliwice, Poland; przemyslaw.struk@polsl.pl; 5 Department of Morphological Sciences, Faculty of Veterinary Medicine, Warsaw University of Life Sciences, 02-776 Warszawa, Poland; wasowiczm@gmail.com

**Keywords:** nitrogen-doped diamond, fiber optic sensor, interferometer, hemoglobin, Fabry–Pérot sensor, biophotnic sensor

## Abstract

In this work we present the fabrication and characterization of a diamond film which can be utilized in the construction of optical sensors for the investigation of biological samples. We produced a nitrogen-doped diamond (NDD) film using a microwave plasma enhanced chemical vapor deposition (MWPECVD) system. The NDD film was investigated with the use of scanning electron microscopy (SEM), atomic force microscopy (AFM) and Raman spectroscopy. The NDD film was used in the construction of the fiber optic sensor. This sensor is based on the Fabry–Pérot interferometer working in a reflective mode and the NDD film is utilized as a reflective layer of this interferometer. Application of the NDD film allowed us to obtain the sensor of hemoglobin concentration with linear work characteristics with a correlation coefficient (R^2^) equal to 0.988.

## 1. Introduction

Among the outstanding properties of diamond, biocompatibility is one of the key functions [1,2], as well as a wide range of optical transparency [3,4]. This set of properties has significant advantages for the development of various sensors, e.g., optical [5], biological, electrochemical [6] sensors. Nanocrystalline diamond (NCD), with a typical grain size of less than 100 nm, has low surface roughness and shows optical properties close to that of single-crystal diamond films [7]. Furthermore, by using doping we can change the morphology of the diamond and therefore its electrical (e.g., electrical conductance [8]) and optical properties (e.g., refractive index [9,10]).

Biocompatibility of diamond is an important property for measuring biological samples [11]. It is worth noting that diamond materials are willingly used by scientists for blood analysis. The red blood cells contain a protein, which forms a red blood dye called hemoglobin (Hb). In living organisms it plays the role of an oxygen transporter [12,13,14,15,16,17]. Hemoglobin is also capable of transporting carbon dioxide (CO_2_) [14,15,16,17]. Some causes of low levels of hemoglobin are anemia, overhydration, chronic bleeding, vitamin B12, iron or folic acid deficiency, bone marrow disorders, chronic diseases (e.g., bacterial, viral, cancer), congenital factors, some medicines (e.g., cytostatics), and ionizing radiation. Elevated hemoglobin is present in original polycythemia (polycythaemia rubra), secondary polycythemia (chronic lung disease, acquired and congenital heart disease, renal tumors), electrolyte disturbances and hypoxia [14,15,16]. Hemoglobin concentration is dependent on age, sex and a number of other factors [14,15,16,17].

Lee and Park [6] used boron doped diamond for electrochemical assay of glucose. The good linear response of glucose oxidation was obtained for a concentration range from 0.5 to 10 mM. Furthermore, this method of glucose assay, when applied to real blood samples, gave results similar to those obtained by commercial methods. Kruusma et al. [18] showed a lead detection in human blood by use of bismuth-film-modified boron doped diamond electrode. The authors achieved a low limit detection of lead in blood up to 10^−8^ mol L^−1^ with excellent inter- and intra- reproducibility and sensitivity. Electrochemical measurements to detect hemoglobin concentration were shown by Zhang and Oyama [19]. The linear relationship between the peak current and the concentration of hemoglobin from 1 × 10^−6^ to 1 × 10^−5^ M was found.

On the other hand, nitrogen is recognized to be a good source for n-type doping in nanocrystalline diamond (NCD) films. Several studies with experimental observations and theoretical predictions were carried out to accomplish effective n-type conductivity in NCD films by adding N_2_ in the plasma during film deposition [20,21,22]. Nitrogen incorporation results in significant mechanical stress and increases the number of vacancy defects due to distortion of the diamond lattice [23]. It has a significant impact not only on electrical properties but also on optical properties of diamond films. Increasing the doping level of nitrogen will affect the refractive index to change from normal to an abnormal distribution, as is observed in metals. In this manuscript we present nitrogen-doped diamond film which was used in the construction of the fiber optic sensor for optical investigation of hemoglobin concentration.

## 2. Experimental Section

### 2.1. Materials and Measurement System

#### 2.1.1. Growth of Nitrogen-Doped Diamond (NDD) Film

The NDD film was deposited on polished n-type silicon substrate (1 cm × 1 cm) by using an ASTeX 6500 (SEKI, Tokyo, Japan) series microwave plasma enhanced chemical vapor deposition (MWPECVD) system. Prior to NDD film growth, the silicon substrate was nucleated with a water based state-of-the-art colloidal suspension of 5 nm detonation nanodiamonds. The NDD film was deposited in a gas mixture of CH_4_ (3%), H_2_ (94%), and N_2_ (3%) with flow rates of 9282, and 9 sccm, a microwave power of 3000 W, a pressure of 65 Torr, and a substrate temperature of 650 °C, respectively. The substrate temperature was measured using a single color optical pyrometer, assuming an optical emission coefficient of 0.3.

#### 2.1.2. Fiber Optic Measurement System

**Figure 1:**
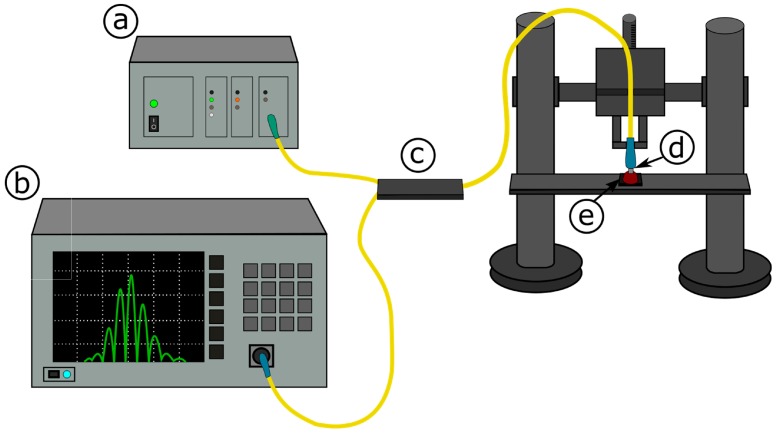
Measurement set-up: (**a**) light source; (**b**) optical spectrum analyzer (OSA); (**c**) coupler; (**d**) measurement head; (**e**) nitrogen-doped diamond film deposited on silicon substrate.

In an experimental set-up, superluminescent diode Superlum Ltd. (Carrigtwohill, Ireland) was used. Parameters of this light source were as follows: S-1550-G-I-20: *λ* = 1550 nm, Δ*λ*FWHM = 45 nm. The detection of the measured signal was performed by using an optical spectrum analyzer (Ando AQ6319, Tokyo, Japan) with resolution bandwidth set to 1 nm. All the devices were connected with single mode fibers (SMF-28, Thorlabs, Newton, MA, USA) and 2 × 1 coupler (Thorlabs, Newton, MA, USA). A set of micromechanical elements for positioning the optical fiber was used in the measurement system (Figure 1). The measurement system was described in detail elsewhere [24].

## 3. Diamond Film Surface and Structure Investigation

**Figure 2:**
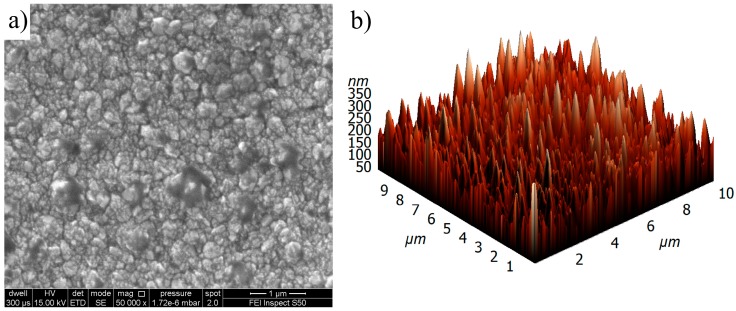
The surface topography of diamond film: (**a**) SEM image; (**b**) atomic force microscopy (AFM) image.

This part of the research was focused on the investigation of surface topography, as well as the structure of nitrogen-doped diamond film. The research of surface topography was focused on the determination of crystalline grain size as well as surface roughness. The research was carried out with the use of two methods: scanning electron microscopy (SEM) (Inspect S50, FEI Company, Hillsboro, OR, USA) and atomic force microscopy (AFM) (N-TEGRA Prima, NT-MDT Company, Moscow, Russia). The resulting SEM image measured with a magnification of ×50000 for nitrogen-doped diamond film is presented in Figure 2a. The presented SEM image confirms that the diamond film is continuous and homogeneous without cracks. The SEM image shows also that the surface of the diamond film is in the form of crystallites with size in the range of ~0.1–0.87 µm.

The investigation of root mean square roughness *R*_q_ of diamond film was carried out by AFM method. The scanning of diamond surface was carried out on a surface area equal to 10 × 10 µm^2^ by HA-NC (High Accuracy NonContact) AFM probe (NT-MDT Company, Moscow, Russia) worked in a semi-contact mode with resonant frequency *f_r_* = 265.42 kHz. The 3D image of surface morphology of investigated nitrogen-doped diamond film is presented in Figure 2b. Analysis of the obtained AFM image showed that the root mean square roughness for nitrogen-doped diamond film is relativity high and equal to *R_q_* = 49.1 nm.

**Figure 3:**
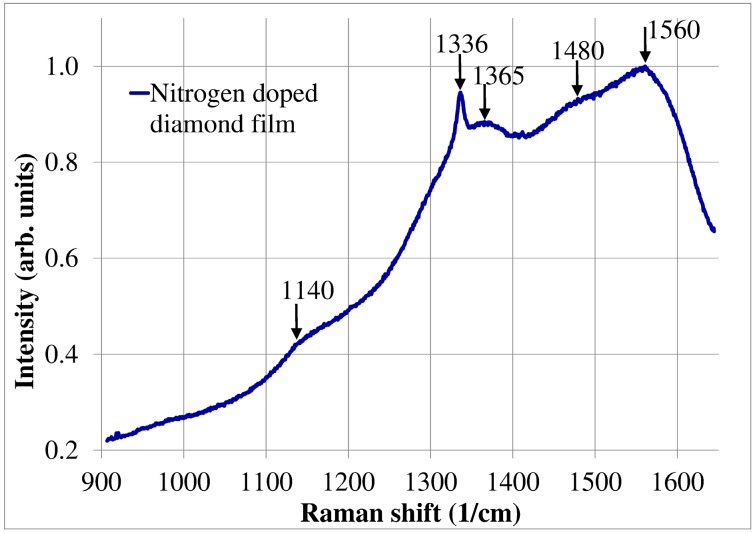
The Raman spectrum of the nitrogen-doped diamond film.

The structure investigation of diamond film was carried out by Raman spectroscopy method. During experiments the N-TEGRA Spectra (NT-MDT Company, Moscow, Russia) measurement setup was used. The diamond film was illuminated by laser with central wavelength *λ_c_* = 532 nm. The Raman spectra of investigated nitrogen-doped diamond film is presented in Figure 3.

The Raman spectrum presented above showed typical signal signatures for the nitrogen-doped diamond film. Five major Raman features at 1140 cm^−1^, 1336 cm^−1^, 1365 cm^−1^, 1480 cm^−1^ and 1560 cm^−1^ are observed from NDD film. The prominent bands at 1365 cm^−1^ and 1560 cm^−1^ are designated as D-band and G-band, respectively, representing the disorder carbon and graphites [25,26,27,28]. The Raman peak for 1336 cm^−1^ (D-band) is characteristic for the diamond phase (sp^3^) [29]. The peak at 1336 cm^−1^ is characteristic for the diamond phase (sp^3^). In the case of nitrogen doped diamond film, the characteristic peak for 1336 cm^−1^ is decreased in comparison to non-doped diamond film. The other Raman features of NDD films at around 1140 cm^−1^ and 1480 cm^−1^ indicate the presence of *trans*-polyacetylene phases locating at grain boundaries [25,27,28]. It is to be noted that the intensity of G-band is increased in comparison to D-band of NDD film as compared to undoped diamond film [30].

## 4. Result

The aim of this research is to investigate if nitrogen-doped diamond film could be used as a reflective layer in Fabry–Pérot interferometer. While using processing in spectra domain in such an interferometer, we can express a signal recorded on spectra analyzer as follows [31]:

(1) $$I_{out}\left(v\right)=S\left(v\right)\left(1+V\cos\left(\Delta \varphi \left(v\right)\right)\right)$$

where: *S*(*v*)—spectral distribution of the light source, *V*—visibility of the signal, Δφ(v)—phase difference between interfering beams.

**Figure 4:**
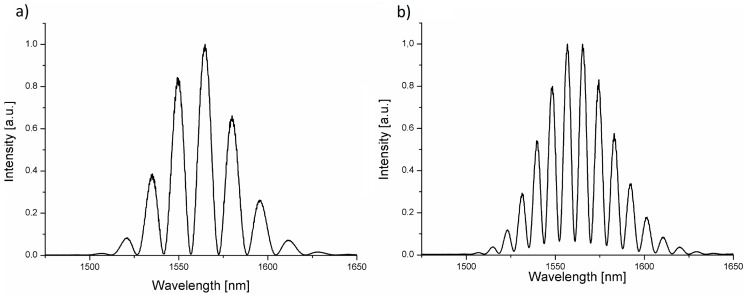
Signal measured with the central wavelength of 1550 nm for nitrogen-doped diamond (NDD) and the length of Fabry–Pérot cavity: (**a**) 80 μm; (**b**) 140 μm.

The phase difference depends [31,32] on an optical path difference (OPD) that is influenced by a geometrical path length and a refractive index of medium. To test if the investigated film can be successfully used as a mirror in the interferometer, we performed 10 series of distance measurements. We changed the cavity length from 0 μm to 200 μm with increment of 20 μm. The signal modulation was observed while changing the cavity length as can be noted in Figure 4. It follows that the interferometer with the NDD film works properly as a distance sensor.

Using recorded spectra we calculated visibility of the signal, which is the most important metrological parameter of the interferometer, by the formula [28]:

(2) $$V=\frac{I_{\max}\;-\;I_{\min}}{I_{\max}\;+\;I_{\min}}$$

where: *V*—visibility of the signal, *I*_max_—maximum intensity of the measured signal, *I*_min_—minimum intensity of the measured signal.

**Figure 5:**
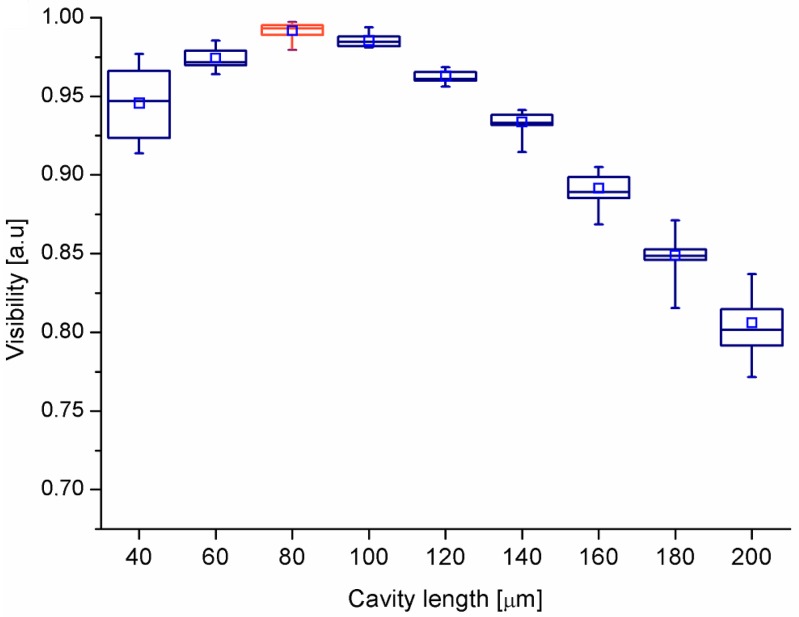
Visibility value as a function of cavity length measured on NDD. The straight lines inside the boxes indicates a median and the squares inside show an average value based on all measurement series.

Figure 5 shows a box plot of signal visibility as a function of cavity length for the investigated film. It shows the distribution of measured data points. The straight lines inside the boxes indicate a median and the squares inside show an average value based on all measurement series.

It can be noted that the signal visibility changes while the cavity length is changing. The visibility of interference signal depends on the optical power was reflected from the two mirrors of the interferometer. The visibility of the fringe is directly related to the distance between these two mirrors, as light beam diverges and is coupled back into the fiber with different efficiency, the influence of the visibility of the measured signal is as described in detail by Milewska et al. [33]. The optimal cavity length where the signal visibility is the highest and the most repeatable equals 80 μm for *λ* = 1550 nm for investigated film. The average value of signal visibility for the optimal cavity length is V = 0.9915.

**Figure 6:**
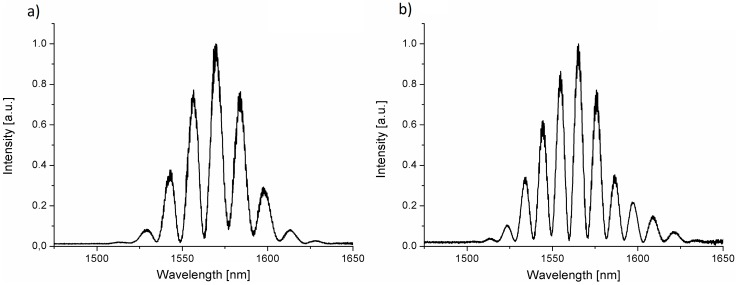
Representative spectra obtained for different hemoglobin concentrations at wavelength 1550 nm on NDD film (**a**) Hb level 7 g/dL; (**b**) Hb level 12.9 g/dL.

For measurements of hemoglobin concentration, we prepared samples with different hemoglobin levels. Figure 6 shows representative hemoglobin spectra obtained for samples with a different concentration of hemoglobin.

**Figure 7:**
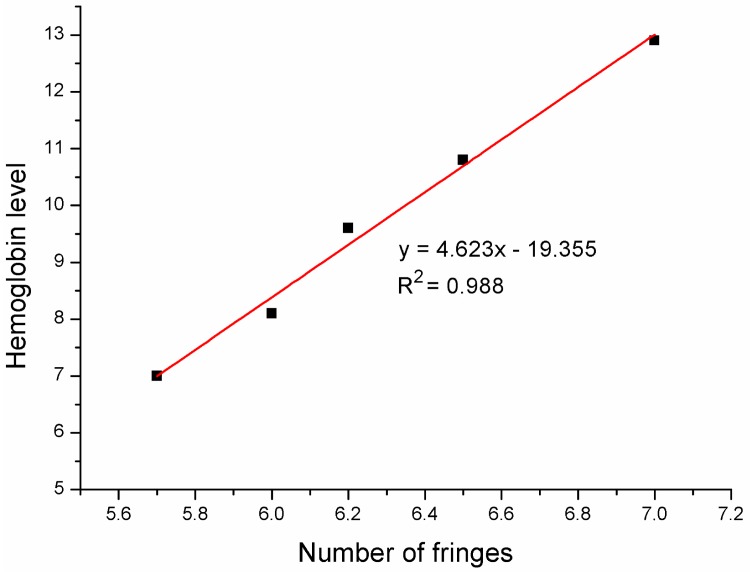
Hemoglobin level as a function of number of fringes—linear fit. Correlation coefficient R^2^ = 0.988.

As can be observed in Figure 7, for the different values of the hemoglobin level in the investigated samples, the spectrum of the measured signal changes. It happens because the change of the hemoglobin concentration influences the refractive index of the sample and therefore the optical phase difference between the interfering beam differs what occurs in the change of maxima number in the measured spectra. This allows us to find the relationship between hemoglobin concentration and the number of maxima in the measured spectrum and propose the model of the relationship between the refractive index vs. hemoglobin level. We counted the number of fringes in spectra for different hemoglobin level in the range of 1525–1600 nm. The results are shown in Figure 6.

It can be seen that the fit is linear. The correlation coefficient R^2^ describes the degree of adjustment of measured data to a theoretical curve. The maximum possible value of R^2^ equals 1 which indicates the ideal fitting. In this case it is equal to 0.988 which means that the data is very well fitted. This follows that nitrogen-doped diamond can be successfully used as a mirror in a Fabry–Pérot sensor for detection of different hemoglobin concentrations.

## 5. Conclusions

The NDD film was deposited on silicon substrate using Microwave Plasma Enhanced Chemical Vapor Deposition (MPE CVD) system with a gas mixture of CH_4_ (3%), H_2_ (94%) and N_2_ (3%). The SEM image shows that the size of the crystallites is in the range of ~0.1–0.87 µm and the AFM image indicates that the *R_q_* is equal to 49.1 nm.

This film was implemented in the construction of the fiber optic sensor of hemoglobin concentration. The sensor shows the ability to perform an optical investigation of hemoglobin concentration with a very good metrological parameters such as visibility value equal to 0.9915 and correlation coefficient equal to 0.988.
